# Brewing with Whole Wheat Bread to Produce Different Beer Styles

**DOI:** 10.3390/foods14101697

**Published:** 2025-05-11

**Authors:** Carlos Martin-Lobera, Jose Fermoso, Carlos A. Blanco, Isabel Caballero

**Affiliations:** 1 Department of Agricultural and Forestry Engineering (Food Technology Area), E.T.S. Agricultural Engineering, University of Valladolid, 34004 Palencia, Spain; 2 Agri-Food Division and Sustainable Processes, CARTTIF Foundation, 47151 Boecillo, Spain

**Keywords:** whole wheat bread, craft beer, lager, weiss, IPA, physicochemical profile

## Abstract

Beer is one of the most widely consumed alcoholic beverages and is rich in nutrients. Meanwhile, bread waste is a major contributor to global food waste. This study investigated substituting up to 50% of malt with whole wheat bread in American lager, Indian pale ale, and Bavarian weiss ale to reduce bread waste and enhance beer’s nutritional profile. The study assessed physicochemical properties, bioactive compounds, and volatile profiles of bread-based beers versus traditional malt-based brews. Results showed that bread beers maintained key properties while increasing bioactive compounds, especially in Bavarian weiss, which had higher total polyphenol content (1.04 mg GAE mL^−1^ compared to 0.507 mg GAE mL^−1^). Antioxidant activity in weiss beer also increased (2.007–2.057 μMol DPPH mL^−1^ relative to 0.68–1.75 μMol DPPH mL ^−1^ in 100% malt weiss). PCA analysis highlighted a distinct bioactive profile in bread beers, with elevated phenylethyl alcohol and ethyl octanoate. Substituting malt with bread was feasible, producing beers of comparable quality and potential health benefits. These findings support bread as a sustainable, cost-effective malt alternative, reducing waste and enhancing beer within a circular economy framework.

## 1. Introduction

Nowadays, food waste is a global problem. Recent studies estimate that 14% of food produced is lost in the supply chain before reaching retail stores [1], while 17% of available food is wasted at marketing levels and consumption [2]. The latest world estimates showed that approximately 931 million tonnes of food waste were generated in 2019, with 61% from households, 26% from food services, and 13% from retail [2].

The excessive production of food to meet the demand of a growing global population, coupled with wasteful consumer behavior, has led to significant food waste, posing a major challenge for sustainability and resource efficiency.

Bread is a very important food in the human diet due to its nutritional composition. For example, 100 g of bread typically contains about 59.8 g of starch, 22.3 g of moisture, 1.56 g of total organic nitrogen, and 8.9 g of protein [3]. It is produced in large quantities to satisfy high consumer demand, mainly in North America and Europe [4]. However, bread has a short shelf life of 3–6 days at room temperature, mainly due to its high nutrient content, making it susceptible to rotting and hardening [5].

This, along with consumer preference for freshly baked goods, has led to bread piling up from bakeries to retailers and homes [6].

The waste hierarchy, developed in the 1970s to prioritize different waste management strategies, has evolved in recent years and has been adapted to food waste. This pyramid prioritizes prevention actions first, followed by routes aimed at the reuse of surplus food suitable for human consumption, the reuse of food not intended for human consumption as animal feed, the recycling of materials in products of high added value (without carrying out complete degradation), nutrient recycling, energy recovery, and, as a last option, the elimination of food waste [7]. Regarding environmental impact, previous studies have examined different management options. For the most part, the results supported the same waste hierarchy. For example, Brancoli et al. [8] evaluated the relative environmental impacts of different treatment options for surplus bread and observed that a reduction at source and the use of surplus bread in different recovery pathways (animal feed, donation, beer and ethanol production) are environmentally preferable options compared to waste (e.g., anaerobic digestion and incineration).

Beer is among the most popular alcoholic beverages in the world [9]. It is typically produced using malted cereal grains (e.g., barley), yeast, hops, and water; however, adjuncts and additives are sometimes used by the brewing companies. Beer is a beverage rich in nutrients such as carbohydrates, amino acids, minerals, vitamins, and polyphenols, which result from a multi-step brewing and fermentation process [10].

Furthermore, it is a beverage rich in antioxidants, such as phenolic compounds, melanoidins, SO₂, and vitamins [11]. Phenolic compounds are a group of chemical substances characterized by the presence of at least one phenolic unit. Several studies have shown that phenols contribute more than 50% to the antioxidant activity of beer [12,13]. Several studies have shown that these compounds, in addition to playing a key role in antioxidant activity (AOX), also affect the sensory stability of beer [14]. Certain polyphenols and their oxidation products are sensory active, affecting beer astringency and bitterness [15,16], and even beer color, aroma, and flavor stability [11,17].

Brewing beer from bread is a viable alternative to traditional beer fermentation, although a significant fraction of malt is required, as malt contains necessary enzymes to break down bread starch into fermentable sugars. Despite the potential use of this bread waste for beer brewing, there are currently few studies on the matter.

Almeida et al. [18] brewed a craft beer with waste bread and concluded that the resulting beer had a 20% lower carbon footprint compared to the control craft beer. Subsequently, Brancoli et al. [8] investigated the use of leftover bread as a substitute for malted barley in brewing (25–28% by weight) and determined the GWP savings. It was concluded that the GWP decreased by 0.46 kg CO_2_ eq. per kg of wasted bread used in brewing. This calculation was obtained without including the reduction in emissions due to the reduced decomposition of bread in landfills. In 2021, Narisetty et al. [19] showed that a maximum of 25% bread can replace barley due to the need for enzymes. Three years later, McDonagh et al. [20] studied the feasibility of using waste bread to brew beer, investigating the impact on alcohol content and the environmental implications of this substitution. The results showed that beer brewed with up to 60% malted barley by weight replaced by bread had sufficient fermentability to produce the required volume of alcohol. They also concluded that the annual carbon footprint was reduced by 7.13% in carbon dioxide equivalent compared to the industrial process.

Recently, in 2025, Dall’Acqua et al. [21] evaluated the possibility of replacing barley malt with wasted bread in ale beer, as well as its impact on the resulting beverage, both during its production and in the final product. The results showed no differences between the control beers and those brewed with bread in most physicochemical and sensory analyses.

In a previous study carried out with different types of bread (wheat, whole wheat, rye, and corn bread), it was demonstrated that up to 50% of malt could be successfully replaced with stale bread, which represents a very important saving for the brewing industry. Furthermore, it was found that beer made with whole wheat bread had a similar physicochemical profile to beer made with 100% malt. In addition, a higher total content of polyphenols and antioxidant capacity was observed, presenting it as a beer with healthier properties and better sensory characteristics than conventional beer [22].

This study is based on our prior research, investigating optimal outcomes achieved in brewing whole wheat bread beers, with the primary objective of evaluating the performance of our brewing process design across different beer styles, specifically, American lager, Indian pale ale (IPA), and Bavarian weiss ale, by comparing the physicochemical characteristics of 100% malt brews versus whole wheat bread-based beers.

## 2. Materials and Methods

### 2.1. Reagents and Standards

Methanol, gallic acid, and Folin–Ciocalteu reagent were purchased from Merck Millipore (Madrid, Spain). 2,2-Diphenyl-1-picrylhydrazyl (DPPH) was acquired from Sigma-Aldrich Química S.A. (Madrid Spain). Sodium chloride (NaCl), sodium hydroxide 0.01 Mol L^−1^, Coomassie blue G.250 (CBBG), D (+)-glucose anhydrous for ACS analysis, and Bradford reagent were obtained from Panreac (Castellar del Vallés, Spain). All solutions were prepared with analytical-grade reagents and distilled water.

### 2.2. Raw Materials

Three different styles of craft beer were elaborated due to their being the most widely consumed beers in the world: American lager for lager beer and, in the case of ale beers, IPA (Indian pale ale) and Bavarian weiss beer. All beers were brewed using 100% malt as control samples. In the experimental beers, 50% of the total malt was replaced with the same weight of whole wheat bread (1:1 substitution). This replacement was done proportionally according to the original recipe, meaning all types of malt were reduced equally, keeping the same proportions as in the control. According to the style, the raw materials used in beer recipes for this study are shown in Table 1.

The yeast strains Saflager S-23, Safale US-05, and Safbrew W-06 were used for the fermentation process in tanks, while the Safale F-2 strain was employed for bottle fermentation; whole wheat bread was acquired in a stale condition (three days after production) from “La Tahona de Sahagún” (Palencia, Spain), a local bread producer, and milling was carried out immediately after its arrival at the laboratory.

### 2.3. Brewing Procedure

All beers were brewed at the University of Valladolid pilot cellar (University of Valladolid Campus, Palencia Spain). Beers (100% malt grains and flakes) were brewed as a control in each style, and total malt amounts added to 10 L of mineral water were 2.381 Kg per American lager, 2.650 Kg per IPA, and 2.170 Kg per Bavarian ale brew. Also, bread beers were made according to the control ones in each cited style, replacing 50% of the weight malt with the same weight of stale whole wheat bread. All beers were brewed in duplicate, in individual 10 L batches, and labeled with abbreviations as follows: 100% malt beers were designated as American lager (LA), Indian pale ale (IPA), and Bavarian weiss ale (W); bread beers were codified the same way as the controls with the addition of the letter “B” at the end.

**Figure 1:**
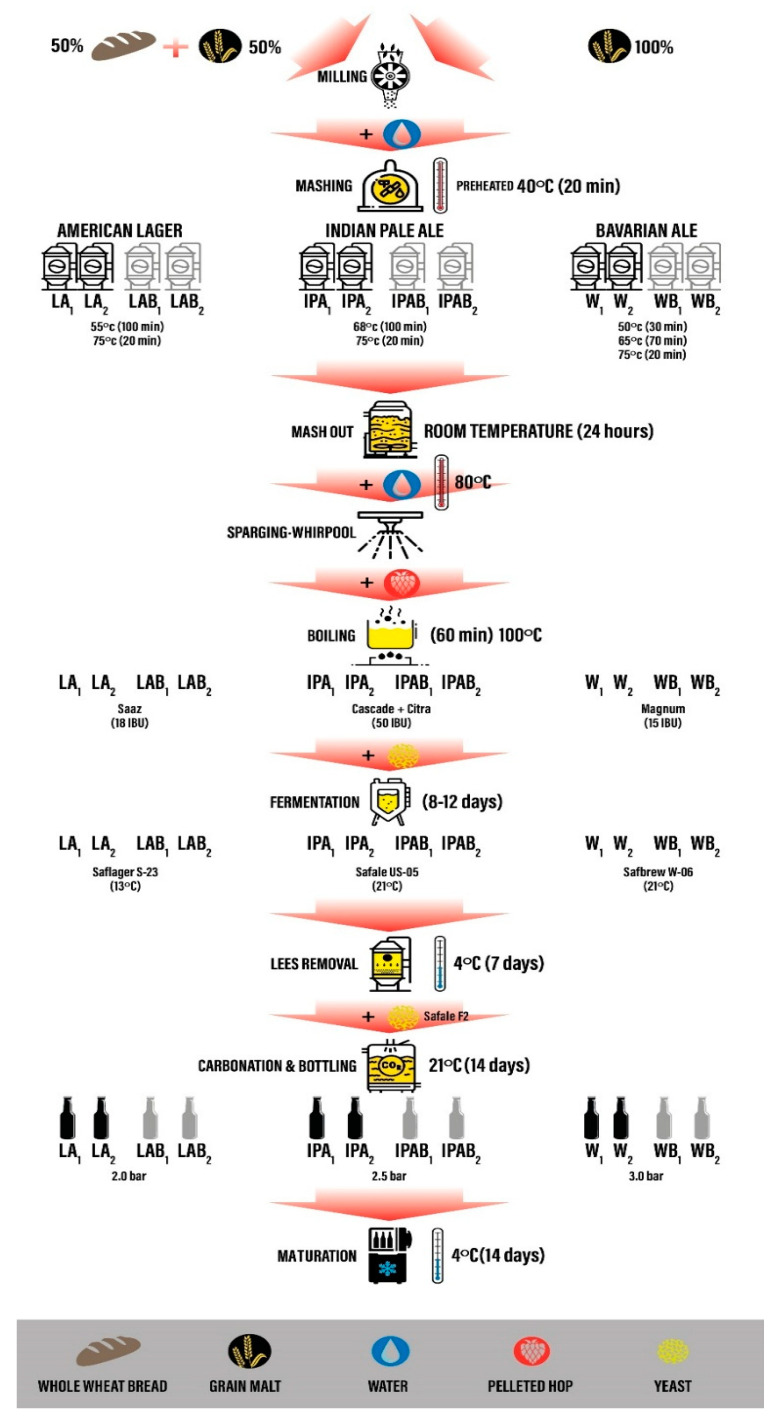
Brewing process diagram.

The detailed brewing process, which was optimized by our team in an early stage and recently published [22], is shown in Figure 1.

First, malts and stale bread were ground separately, in a two-roll mill spaced 1 mm, just before mashing. Second, the mineral water was preheated to a temperature of 40 °C, and the resulting ground malt or ground malt plus bread at 50% by weight were poured into buckets and stirred for 20 min. Next, the temperature was increased at different steps, depending on each recipe, as described in Figure 1, and kept for 24 h for extended maceration at room temperature in order to prolong enzymatic activity and improve starch and protein hydrolysis, thereby enhancing fermentable sugar availability. Then, after lautering wort was transferred to the kettle, the remaining bagasse was sparged using hot mineral water at 80 °C until the final volume of 10 L was completed. All batch wort were boiled at 100 °C for 60 min. Saaz pelleted hop was added at the start of boiling to obtain 18 International Bitterness Units (IBU) for the American lager recipe. In the case of Indian pale ale, two different pelleted hops were used: Cascade hop was infused to contribute 35 IBU at the beginning of boiling, and Citra hop was added in the middle of this procedure to achieve the remaining 15 IBU. For the Bavarian ale recipe, Magnum pelleted hop was introduced at the start of boiling to achieve 15 IBU. After boiling, the hot trub was removed, and the wort was rapidly cooled using a freezing chamber at −25 °C to reach the appropriate fermentation temperature (Figure 1). Before yeast inoculation, all wort batches were oxygenated by manual agitation for 2 min to ensure proper yeast activity.

Different commercial yeasts were used for primary fermentation, applied at a concentration of 0.5 g per liter, according to Figure 1. All dry yeast strains were rehydrated prior to inoculation. Rehydration was performed in sterile water at 25 °C, using a yeast-to-water ratio of 1:10 (*w*/*v*), in accordance with the manufacturer’s guidelines. The suspension was allowed to rest undisturbed for 15 min before being pitched into the wort. Primary fermentation was conducted in 10 L stainless steel tanks for 8 to 12 days. The tanks were equipped with an overpressure valve to allow safe CO₂ release. Fermentation temperature was consistently maintained by placing the tanks in a climate-controlled laboratory room, set to the specific conditions required for each beer style (13 °C for lager and 21 °C for ale fermentations). After beers reached the final attenuation degree, the temperature was gradually reduced to 4 °C in a cooling chamber; the beer remained in the tanks for a week to facilitate the next stage of lees removal and beer maturation. After lees removal, all containers were tempered to 21 °C. Carbonation was then carried out in 330 mL glass bottles using dextrose and Safale F-2 yeast. Dextrose was added at 4 g per liter of beer per bar of target pressure, aiming to reach internal CO₂ levels of 2.0 bar for lager, 2.5 bar for IPA, and 3.0 bar for Bavarian beer. Then, all bottles rested for 14 days to finish fermentation at 21 °C and final pressure was verified using crown-cap aphrometers. Finally, all beer bottles matured in a refrigerated chamber at 4 °C for 2 weeks.

### 2.4. Physicochemical Analysis

Unfiltered samples were extracted, excepting those used for color analysis, in which samples were previously centrifugated in a centrifuge (Bunsen, model KOCH 1460, Humanes de Madrid, Spain) at 4000 rpm for 5 min, as well as filtered by a vacuum filter (model: Kitasato) and Millipore filters (Merck Millipore, Darmstadt, Germany) of 0.45 microns.

- pH was measured with a pH-meter (HACH-LANGE, calibrated sensiON™^+^ pH3 model, Hospitalet, Spain).
- Acidity: pH-meter measurements were taken continuously. An acid–base titration was performed until a pH of 7 was reached. Then, the results were expressed in terms of lactic acid percentage.
- Turbidity was measured with a turbidimeter (Hanna Instruments, HI 98703 model, Eibar, Spain). The different samples were placed in a transparent glass container with a lid, and then each container was placed in the turbidity meter to obtain NTU turbidity values (Nephelometric Turbidity Units).
- Alcohol By Volume (ABV) was measured with an ebulliometer (GAB system, 1010006 model, Moja, Spain). First, it was calibrated with distilled water as a standard. Subsequently, the boiling temperatures of the standard (water) and the test sample (beer) were compared, and the volumetric alcohol content was calculated to the nearest 0.1 ABV using a ruler scale. Full attenuation of each batch was verified by three consecutive daily density readings at 20 ± 0.1 °C (stable FG = 1.010–1.012 SG) using a calibrated densimeter. The ebulliometric values were then validated by comparing the resulting % ABV with those calculated from the original and final gravities according to the EBC reference equation.
- Real Extract was determined in accordance with the principles of Analytica-EBC (Real Extract of Beer) by direct gravimetry. After degassing each beer at 20 ± 0.1 °C, an accurately weighed 1.000 ± 0.005 g aliquot was transferred to a pre-dried, tared pan in a thermobalance (Gibertini Eurotherm, Novate Milanese, Italy). The sample was dried at 105 °C until the rate of mass loss was <0.1 mg per 30 s, guaranteeing constant weight. The residue represents the real extract, expressed as % (m/m).

### 2.5. Spectrophotometric Analysis

Samples were first decarbonated by magnetic stirring for 30 min, and then centrifuged in a centrifuge (Bunsen, model KOCH 1460, Humanes de Madrid, Spain) at 4000 rpm for 5 min, as well as filtered by a vacuum filter (model: Kitasato) and Millipore filters (Merck Millipore, Madrid, Spain) of 0.45 microns.

All filtered beers were measured on a spectrophotometer (ThermoFisher Scientific, model 20 Genesys UV-Vis, Madrid, Spain).

- Color (EBC): Beer color was determined according to the standard method of the European Brewery Convention (EBC).
- Total polyphenol content (TPC): The total polyphenol content was determined by the Folin–Ciocalteu method by measuring absorbance at 760 nm [23], using the spectrophotometer mentioned above. A calibration line was performed using different concentrations (0.0–30 mg L^−1^) of standard solutions of gallic acid, resulting in the following equation: Y = 0.0243x + 0.0209, R = 0.9959. The concentration of total phenols is expressed as mg of GAE mL^−1^ of the sample.
- Antioxidant capacity (DPPH): The antioxidant capacity of the beer samples was determined according to the method described by Abderrahim et al. [24]. Beer samples, once filtered and diluted (the 50 μL sample or the blank control), were introduced and mixed with 1000 μL of DPPH (60 μMol L^−1^ dissolved in methanol 1: 1/10 mMol L^−1^ Tris-HCl buffer pH 7.5) in a 5 mL volumetric flask. At 0 min, and after 20 min of incubation at room temperature in the laboratory (21 ± 2 °C), a small volume was introduced into 10 mm quartz cuvettes, and absorbance was measured at 520 nm with the spectrophotometer mentioned above. The antioxidant capacity of the beer, expressed in μMol DPPH mL^−1^, was calculated using the following mathematical formula:
  $$µMol\;(DPPH\;{mL}^{-1})\;=\;((A_{0}\;-\;A_{t})/A_{0})\;\times \;((V_{t}\;[DPPH]\;\times \;FD)/mL)$$
  where *A*_0_: control absorbance (DPPH diluted in methanol); *A_t_*: sample absorbance; *V_t_*: total reaction volume in liters; [*DPPH*]: *DPPH* concentration; *FD*: dilution factor; and mL: sample milliliters used in the reaction.
- Protein content: A method based on the Bradford test [25] was followed, according to which the quantification of proteins is based on the union of the Coomassie blue dye G-250 (Bradford reagent) with the proteins available from the analyzed beer samples. In these analyses, 3140 μL of distilled water and 200 μL of the Bradford reagent were added to 60 μL of the beer sample in a test tube. A calibration line from 1 to 40 μL was also constructed using serum albumin (0.1 μg μL^−1^). Finally, samples were measured at 595 nm.

### 2.6. Headspace Gas Chromatography–Mass Spectrometry Analysis (HS-GC-MS)

Sample preparation was performed according to the general method described by Liu et al. [26].

Samples were analyzed by HS-GC-MS (headspace gas chromatography coupled to a mass spectrometer) in a QP2010 Shimazdu device with an AOC 5000 autosampler (Shimadzu Europa GmbH, Duisburg, Germany) and an HP-5MS column (30 m long, 0.25 mm internal diameter, and 25 μm of film).

Two mL of each previously filtered beer were placed in a 10 mL HS vial with NaCl 20% (*w*/*v*), and heated up to 80 °C at 250 rpm for 15 min; this was performed prior to the 100 μl HS injection of the sample in splitless mode.

The pressure was set at 110 kPa and Helium was used as a carrier gas. The interface temperature was 250 °C and the injector temperature was 120 °C. The oven followed the following program: an initial temperature of 40 °C for 2 min, a ramp-up of 10 °C/min to 140 °C, and a second ramp-up of 7 °C/min to 250 °C. Data were acquired in full scan mode in a m/z range of 30–350, and peak identification was determined by comparison with the NIST08 and WILEY229 libraries.

### 2.7. Statistical Analysis

All analyses were performed in triplicate. Statistical analysis was carried out using Xlstat v.2023.3.1 statistical software (Addinsoft, Paris, France).

Data analysis was conducted to identify differences between the mean values for physicochemical and spectrophotometric measurements; analysis was performed using an analysis of variance (one-factor ANOVA) and Tukey’s significant difference test (HSD), with statistical significance being set at a *p*-value < 0.05.

Principal component analysis (PCA) was used for HS-GC-MS and physicochemical analyses.

## 3. Results and Discussion

### 3.1. Physicochemical Analysis

The main physicochemical properties of beer are shown in Table 2.

Using whole wheat bread as a partial malt substitute in brewing did not result in significant changes in turbidity, except for hazy beers like Bavarian weiss styles, where a decreasing trend and significant differences were observed when malt was replaced by whole wheat bread versus the control. In fact, high turbidity is a distinguishing feature of wheat beers due to their high molecular weight proteins and polysaccharides [27].

These results are consistent with our previous research, which showed that bread decreased turbidity when used as a starchy source in pale ale beers [22], perhaps due to the reduction in malt content caused by its partial replacement.

The pH values ranged from 3.78 to 4.42. No significant differences in pH were observed between bread beers and 100% malt beers among ale styles. Indian pale ales had higher values (4.20–4.42) compared to the other beers; this may be attributed to the use of a greater amount of hops compared to the other styles.

Significant differences were observed between the control American lager (LA) and the American lager bread beer (LAB), where the pH increased with the addition of the whole wheat bread. Habschied et al. [28] studied 26 samples of different beer styles and reported pH values of 3.9–4.12 for lagers and 3.62–4.64 for Indian pale ales, which are in range with the values obtained in this study.

As for the acidity, no statistically significant differences were found in the samples, regardless of beer style or raw material used. However, the weiss beers showed higher acidity; this was also observed in the study of Pai et al. [29], where the sample of lager beers with the lowest pH was also the most acidic. Moreover, pH and acidity are considered crucial criteria in the brewing industry, as they influence sensory parameters such as color, taste, and the biological and chemical stability of the beer [29,30]. The results obtained in this research showed that samples presented lower acidity in general compared to Silva et al. [30], who studied craft beers, where lagers had an average acidity of 0.18%, Indian pales ales 0.28%, and weiss beer 0.17%.

All brewing styles, independent of the raw material, achieved the alcoholic strength recommended by the Brewers Association Beer Style Guidelines [31], and Indian pale ales showed the highest ABV, ranging from 6.70–7.0%. Byeon et al. [32], who studied wheat malt beers, reported an alcohol content of 4.83–5.37%; these results are similar to those obtained in our investigation for weiss and weiss bread beers. Another study reported ranges of alcohol content in the samples used of 3.2–6.7% for American lagers, which agrees with our findings [33]. All these results confirm that the alcoholic content was consistent with the expected parameters, regardless of the partial substitution of malt with whole wheat bread in each beer style.

In general, all beers showed no significant differences in real extract content, except for Indian pale ales, which presented higher values (6.48–6.74%). This trend may be related to the formulation of IPA recipes, which typically involve higher malt concentrations and dry hopping, both of which may contribute to an increased level of non-fermentable substances in the final beer. A similar observation was reported in previous research on bread beers, which showed lower real extract values compared to beers brewed with 100% malt. This trend may be attributed to the fact that, in the absence of bread, malt can release more organic and fermentable compounds into the wort during mashing [22].

Finally, the physicochemical parameters analyzed in our investigation were consistent with the recent findings in 2025 of Dall’Acqua et al. [21], who studied wheat craft beers brewed with wasted wheat bread as a partial starchy adjunct. They reported no significant differences in ethanol concentration between control and bread-enriched beers. Likewise, both studies observed similar pH values, reduced turbidity, and a slight decrease in real extract when bread was used as a partial malt substitute.

### 3.2. Spectrophotometric Properties

#### 3.2.1. Color (EBC)

**Figure 2:**
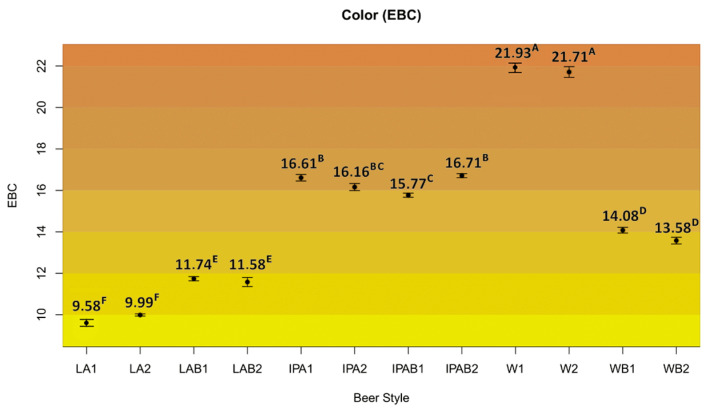
Color measurements in the craft bread beers studied. Different uppercase letters mean significant differences (*p* < 0.05).

The beer color results were heterogeneous. American lagers had the lowest EBC (9.58–11.58), displaying their characteristic golden tone. Significant differences were observed in lager and weiss beers when the control beers versus bread beer samples were compared, although this effect was divergent, increasing in lager bread beers and decreasing in weiss bread beers (Figure 2). The higher hue observed in the control weiss beers may be attributable to their substantially higher residual turbidity, which can scatter light and artificially raise the absorbance at 430 nm, thereby increasing the calculated EBC value.

These results indicated that bread contributes to a darker color when pale malts are used, like in the American lagers, but that the opposite effect is observed when compared to roasted malts, being unable to achieve the same EBC as these kinds of malts. This could be due to the pigments of whole wheat bread in the crumb and especially in its brown crust, which influences the wort and finished beer darkness.

#### 3.2.2. Total Polyphenol Content (TPC)

Polyphenolic compounds in beer typically derive from hops (30%) and malts (70%) or develop during the chemical reactions that take place in the brewing process [34]. Important characteristics, such as astringency, body, mouth fullness, and flavor, are influenced by polyphenols. The presence of these components is of great interest to the industry because they prevent oxidation and affect colloidal and foam stability [15,35]. Furthermore, the presence of phenolic compounds is attributed to positive health impacts when beer consumption is moderated [36].

**Figure 3:**
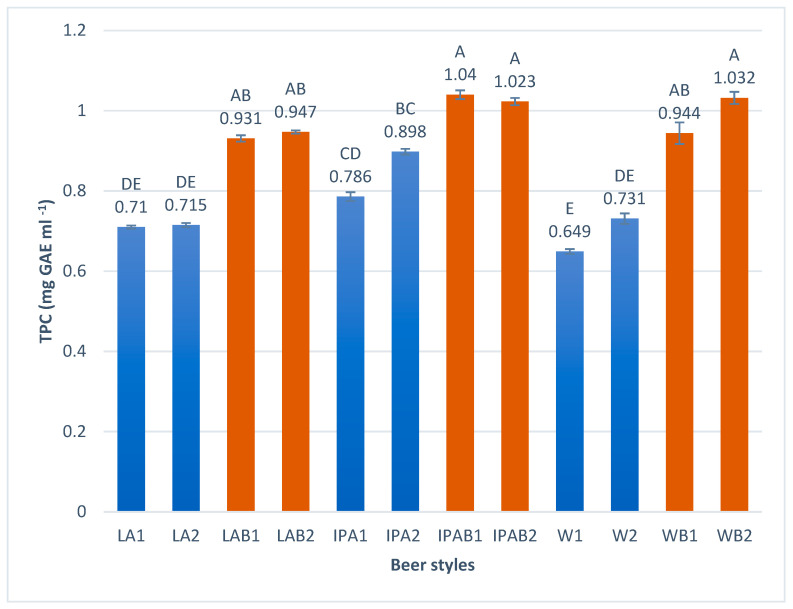
Total polyphenol content in the craft bread beers studied. Different capital letters indicate significant differences (*p* < 0.05).

The TPC result for the samples, as shown in Figure 3, ranges from 0.649 to 1.04 mg GAE mL^−1^. A significant increase in the total polyphenol content was observed in bread beers, regardless of the style. The highest result was found in the sample IPAB1 with 1.04 mg GAE mL^−1^, and the lowest in W1 with 0.649 mg GAE mL^−1^.

The findings in our study for lager and weiss beers, particularly those brewed with bread, were higher than the values reported by Bertuzzi et al. [36], who analyzed TPC in 80 samples of craft and industrial beers, showing the average values of 0.507 mg GAE mL^−1^ for craft lagers and 0.403 mg GAE mL^−1^ for wheat craft beers.

Nardini and Foddai [37] reported TPC ranging from 0.274 to 0.321 mg GAE mL^−1^ for lagers and 0.383 to 0.446 mg GAE mL^−1^ for ale-style beers. Habschied et al. [35] examined commercial lager brands, reporting TPC results from 0.076 to 0.117 mg GAE mL^−1^, and Piazzon et al. [38] reported an average value of 0.452 mg GAE mL^−1^ for lager, 0.484 mg GAE mL^−1^ for Pilsner, 0.504 mg GAE mL^−1^ for wheat, and 0.563 mg GAE mL^−1^ for ale beers. Silva et al. [30] determined that the TPC for Indian IPA craft beers ranged between 0.514 and 0.936 mg GAE mL^−1^. Additionally, Iannone et al. [39] reported a lower TPC for craft IPA. All previously reported values are lower than the TPC determined in this study, particularly in comparison with whole wheat bread beers.

Although the TPC ratio largely depends on the beer style, we discovered an important fact. All the values obtained in whole wheat beers were higher than the controls. So, the use of whole wheat bread significantly increased the total polyphenol content. The fermentation process that wheat undergoes during bread making, as well as maceration and fermentation during beer production, allows the polyphenols present in wheat to become bioavailable [34,40,41]. This could explain the increase in these parameters.

#### 3.2.3. Antioxidant Activity by DPPH

**Figure 4:**
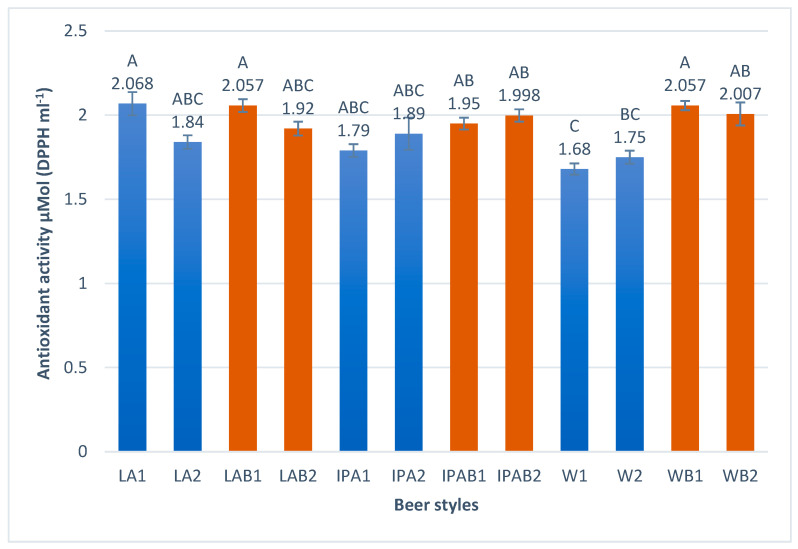
Antioxidant activity in the craft bread beers studied. Different capital letters indicate significant differences (*p* < 0.05).

The results presented in Figure 4 show the differences in antioxidant activity among the samples analyzed.

Despite replacing malt with whole wheat bread by up to 50%, the resulting beers did not lose antioxidant capacity; on the contrary, they showed slightly higher results compared to the control beers. This effect is particularly noticeable for weiss beers, where beers brewed with bread ranged from 2.007–2.057 $\mu Mol\;DPPH{mL}^{-1}$ compared to 1.68–1.75 $\mu Mol\;DPPH{mL}^{-1}$ for the 100% malt beers. Mitić et al. [42] studied commercial beers, in which lower values for antioxidant capacity than those we obtained were reported, ranging from 0.56 to 1.66 $\mu Mol\;DPPH{mL}^{-1}$.

#### 3.2.4. Protein Content

**Figure 5:**
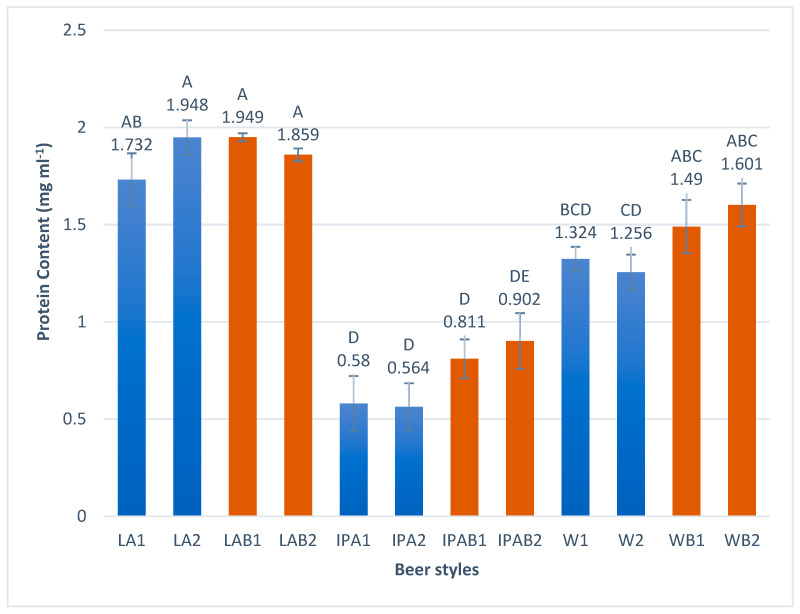
Protein content in the craft bread beers studied. Different capital letters indicate significant differences (*p* < 0.05).

Figure 5 shows the protein content obtained for the different samples; the values range from 0.564 for control IPA beers to 1.949 $mg\;{mL}^{-1}$ in bread lager beers. The results showed that the whole wheat bread beers had protein levels comparable to the 100% malt beers, with a slight increase observed in the bread brews for IPA and weiss styles. Hu et al. [43] reported a higher protein content in wheat craft beer in comparison to 100% malt control. Protein plays a key role in the foam stability, mouthfeel, and overall quality of the beer [44]. It is also involved in the formation of haze in beer, which affects its sensory characteristics [45].

**Figure 6:**
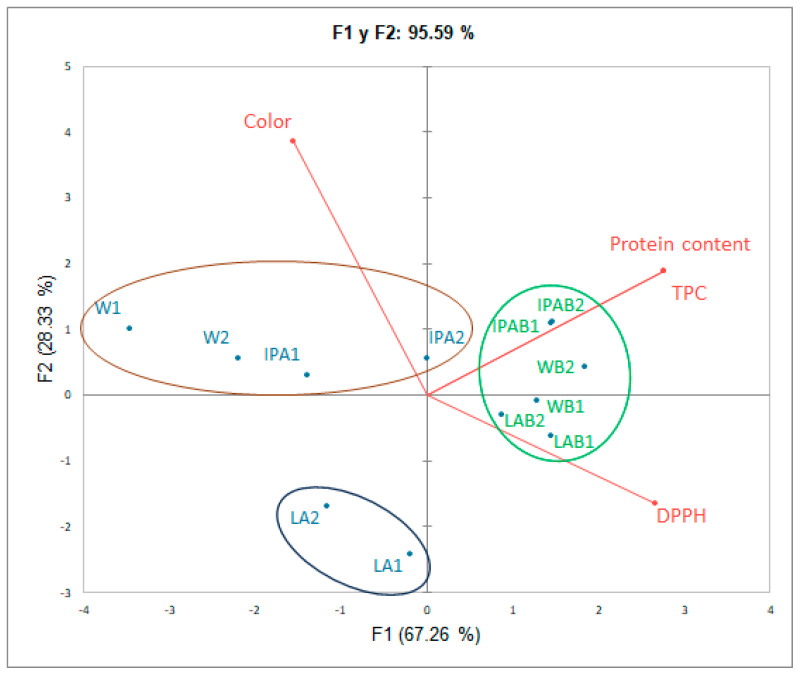
Principal Component Analysis of the bioactive compound profile in craft beers.

To analyze the differences in color and bioactive compounds, like TPC, Antioxidant Capacity, and Protein Content, the Principal Component Analysis (PCA) could effectively screen feature components to distinguish different samples by reducing data dimensionality, thereby identifying more understandable features and accelerating the processing of valuable sample information [46]. PCA provided a comprehensive visualization of the relationship between the samples and their variables, capturing 95.59% of the total variance through two principal components: F1 at 67.26% and F2 at 28.33% (Figure 6). The samples of this study clustered into three groups: the first group (second quadrant) includes the control weiss and IPA control beers, the second group (first and fourth quadrants) comprises the samples made with whole wheat bread, and the third group (third quadrant) comprises the control lagers. These groupings suggest close relationships among samples within each group, indicating similar characteristics.

The first principal component (F1) explains most of the variance, highlighting the importance of protein content, TPC, and DPPH. Samples brewed with bread (LAB, IPAB, WB) exhibit higher values for these variables, suggesting enhanced nutritional and antioxidant profiles. The co-occurrence of TPC and DPPH vectors in the first component (F1) indicates a close relationship between these variables, as shown in previous studies, which have reported a significant correlation between these parameters [37], attributing the antioxidant activity of beer mainly to its phenolic compound content [47]. These associations were likewise apparent in the findings of our study, where the samples made with whole wheat bread (LAB, IPAB, and WB) showed a tendency toward higher antioxidant activity, while presenting higher values of TPC. The color variable, which correlates positively with F2, further differentiates the samples, particularly the lager control beers, which exhibited the lowest color values, and the weiss control beers, which displayed the highest.

### 3.3. Headspace Gas Chromatography-Mass Spectrometry (HS-GC-MS) Detection

The main volatile compounds present in beers were identified and relative quantification was expressed as a percentage (%) by comparing the area under each individual peak to the total chromatographic peak areas. Among the 11 volatile compounds identified, only eight were included in the volatile profile due to their higher relative areas. Furthermore, no off-odor volatile compounds (e.g., vicinal diketones or staling aldehydes) were detected in our HS-GC-MS analyses, with concentrations below the detection limits of the method. The results for these eight predominant volatile compounds, including their retention times and peak area percentages (%), are presented in Table 3.

The difference in flavor beer compounds may be caused by the raw materials used, technological parameters, and yeast [48,49]. Beer flavor is affected by various volatile organic compounds, including alcohols, esters, aldehydes, ketones, and phenols [50]. These compounds were also identified by other researchers using the GC–MS technique [27,51], specifically in Chinese craft IPA and Pilsner lager beer [52], showing significant levels of isoamyl acetate, phenylethyl alcohol, various ethyl compounds, and minor concentrations of compounds like acid ethyl esters [27].

**Figure 7:**
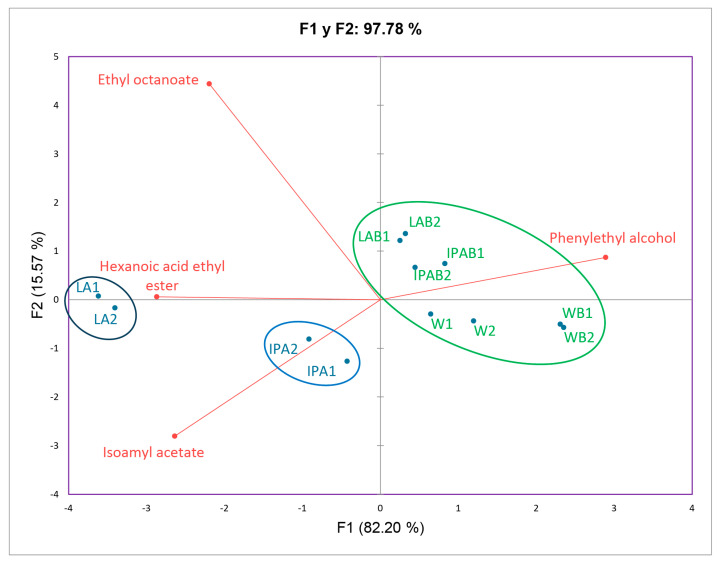
Principal component analysis of volatile compounds in different craft beer styles.

Principal Component Analysis (PCA) was used to analyze data trends, focusing on the key compounds. Isoamyl acetate, phenylethyl alcohol, ethyl octanoate, and hexanoic acid ethyl ester were then selected as key volatile compounds to group the different beers, as they each contributed more than 8% of the relative areas. Two principal components were extracted, explaining 97.78% of the total variance. F1 accounted for 82.20% of the total variance, while F2 explained an additional 15.57%. A scatter plot (biplot) of the analyzed beers was generated by plotting F1 against F2 (see Figure 7). Notably, the “isoamyl acetate” variable was found to be independent of other variables, showing negative values for both Factor 1 and Factor 2, as was the case for phenylethyl alcohol, which exhibited positive values in both factors.

The other two main volatile compounds, hexanoic acid ethyl ester and ethyl octanoate, displayed negative loadings in F1 and positive values in F2.

Based on the PCA results and the beer samples studied (Figure 7), bread beers and weiss beers were grouped together. These beers were all made with wheat as the raw material, either in malt or whole bread form, and were characterized by higher levels of phenylethyl alcohol, particularly in weiss bread beers. This led to pronounced fruit and floral aromas like rose and honey, with a taste profile including apricot notes, typical of wheat beers. These results were consistent with a recent investigation among Chinese beers, which detected significant values in phenylethyl alcohol contents, making them significant compounds for beers [52]. Moreover, a recent study conducted in 2024 by Coelho et al. [53] on beers brewed with a 50% replacement of malt by stale bread also detected 2-phenylethanol, an alcohol known to contribute a rose-like fragrance. Also, in accordance with our earlier investigation, beers brewed using whole wheat bread exhibited a distinctive presence of ethyl octanoate [22], particularly observed across lager and IPA styles.

## 4. Conclusions

Our results indicate that beers brewed with whole wheat bread as a partial malt substitute showed comparable quality to control beers (100% malt) across most physicochemical parameters. Notably, all beers achieved the recommended alcoholic strength, regardless of beer style, and the lager beers showed higher color intensity, attributed to the use of bread. Furthermore, whole wheat bread was found to reduce turbidity during brewing, which was particularly evident in cloudy beer styles such as Bavarian weiss.

Despite replacing up to 50% of malt with whole wheat bread, the resulting beers maintained their antioxidant capacity and protein content and, in general, showed slightly higher values than the control beers, especially evident in weiss beers. Moreover, a significant increase in total polyphenol content was observed in bread beers of all styles.

In addition, a characteristic volatile profile including phenylethyl alcohol and ethyl octanoate was identified in all wheat beers, even when malt was partially replaced by whole wheat bread.

Overall, our findings suggested that using whole wheat bread as a partial substitute for malt added nutritional value due to the health benefits of polyphenol compounds and their antioxidant activity, which could be beneficial for moderate consumers. Furthermore, these results present an opportunity to study the shelf-life behavior of whole wheat bread beers, especially in view of the observed increases in polyphenols and antioxidant capacity compared to 100% malt beers.

Finally, our results confirm that it is feasible to replace up to 50% of malt with whole wheat bread for brewing. Whole wheat bread provided comparable physicochemical properties and bioactive benefits across all beer styles compared to the control beers.

These results represent a significant advancement for the beer industry, reducing malt costs and enabling the reuse of waste bread.

## Figures and Tables

**Table 1:** Brewing raw materials used in the elaboration of craft beers.

Raw Material	American Lager Beer	IPA Beer	Bavarian Weiss Beer
Grain malt and flakes (% by weight)	Pale Ale EBC 7 (Castle Malting, Beloeil, Belgium) (85%)	Pilsner EBC 3 (Weyermann, Bamberg, Germany) (81%)	Pilsner EBC 3 (Weyermann, Bamberg, Germany) (50%)
	Corn flakes EBC 3.5 (Castle Malting, Beloeil, Belgium) (10%)	Munich Type I EBC 12 (Weyermann, Bamberg, Germany) (15%)	Pale Wheat malt EBC 5 (Weyermann, Bamberg, Germany) (50%)
	Rice flakes EBC 2.5 (Castle Malting, Beloeil, Belgium) (5%)	Cara amber EBC 60 (Weyermann, Bamberg, Germany) (4%)	
Pelleted hops	Saaz 3.80% a.a. (Laguilhoat, Fuenlabrada, Spain)	Cascade 6.80% a.a. (Laguilhoat, Fuenlabrada, Spain)	Magnum 13.10% a.a. (Laguilhoat, Fuenlabrada, Spain)
		Citra 12.70% a.a. (Laguilhoat, Fuenlabrada, Spain)	
Bread	Whole wheat	Whole wheat	Whole wheat
Yeast	Saflager S-23 (Fermentis, Marcq-en-Baroeul, France)	Safale US-05 (Fermentis, Marcq-en-Baroeul, France)	Safbrew W-06 (Fermentis, Marcq-en-Baroeul, France)
	Safale F-2 (Fermentis, Marcq-en-Baroeul, France)	Safale F-2 (Fermentis, Marcq-en-Baroeul, France)	Safale F-2 (Fermentis, Marcq-en-Baroeul, France)
Water	Monte Pinos (Carbónicas Navalpotro S.A, Almazán, Spain)		

**Table 2:** Values of physicochemical properties in craft beers (mean ± S.D).

Sample	Turbidity (NTU)	pH	Acidity (% Lactic Acid)	ABV (%)	Real Extract (%)
LA1	620.33 ± 52.29 ^CD^	3.92 ± 0.01 ^D^	0.03 ± 0.02 ^A^	5.52 ± 0.10 ^B^	5.65 ± 0.24 ^DE^
LA2	684.33 ± 16.01 ^D^	3.94 ± 0.01 ^D^	0.04 ± 0.01 ^A^	5.56 ± 0.04 ^B^	5.65 ± 0.13 ^DE^
LAB1	542.00 ± 18.73 ^DE^	4.16 ± 0.01 ^BC^	0.04 ± 0.01 ^A^	5.25 ± 0.10 ^B^	6.15 ± 0.21 ^BC^
LAB2	462.00 ± 9.00 ^E^	4.12 ± 0.01 ^C^	0.04 ± 0.01 ^A^	5.43 ± 0.15 ^B^	5.73 ± 0.30 ^CD^
IPA1	675.67 ± 26.5 ^D^	4.42 ± 0.02 ^A^	0.04 ± 0.01 ^A^	6.98 ± 0.13 ^A^	6.74 ± 0.16 ^A^
IPA2	627.33 ± 29.19 ^CD^	4.31 ± 0.09 ^AB^	0.04 ± 0.01 ^A^	7.00 ± 0.10 ^A^	6.48 ± 0.09 ^AB^
IPAB1	620.00 ± 5.29 ^CD^	4.31 ± 0.02 ^AB^	0.04 ± 0.01 ^A^	6.70 ± 0.22 ^A^	5.93 ± 0.13 ^CD^
IPAB2	671.67 ± 37.07 ^D^	4.20 ± 0.04 ^BC^	0.04 ± 0.01 ^A^	6.72 ± 0.19 ^A^	5.99 ± 0.15 ^CD^
W1	1023.67 ± 39.70 ^A^	3.85 ± 0.03 ^D^	0.05 ± 0.01 ^A^	5.37 ± 0.15 ^B^	5.46 ± 0.11 ^DEF^
W2	1017.33 ± 30.35 ^A^	3.84 ± 0.02 ^D^	0.05 ± 0.01 ^A^	5.23 ± 0.15 ^B^	5.21 ± 0.16 ^EF^
WB1	807.67 ± 27.50 ^B^	3.78 ± 0.01 ^D^	0.05 ± 0.01 ^A^	5.22 ± 0.10 ^B^	5.17 ± 0.12 ^EF^
WB2	867.67 ± 16.44 ^B^	3.82 ± 0.01 ^D^	0.05 ± 0.01 ^A^	5.40 ± 0.10 ^B^	5.06 ± 0.05 ^F^

^A–F^ Means without any common letter within the same column are significantly different (*p* < 0.05).

**Table 3:** Relative main peak areas (%) of volatile compositions in craft bread beers.

Sample	Isoamyl Acetate	Butyl Alcohol	Hexanoic Acid Ethyl Ester	Glycine Benzoil	Phenylethyl Alcohol	Ethyl Octanoate	Phenethyl Acetate	Decanoic Acid Ethyl Ester
Retention Time (min.)	5.33	7.39	7.55	9.23	9.53	10.87	11.84	13.92
LA1	54.00	3.09	8.78	1.19	12.18	15.79	1.78	3.19
LA2	52.90	2.89	8.99	1.56	13.77	14.31	1.63	3.96
LAB1	24.19	2.17	4.93	5.70	47.54	12.57	1.89	0.99
LAB2	22.98	1.80	5.01	5.45	49.42	12.87	1.80	0.67
IPA1	41.19	4.70	5.42	5.60	32.85	5.60	2.58	2.06
IPA2	40.97	3.94	6.05	6.50	29.54	7.84	2.78	2.38
IPAB1	25.73	2.21	3.07	6.18	47.69	10.75	2.06	2.31
IPAB2	27.72	1.89	4.12	5.34	46.97	10.86	1.33	1.77
W1	29.94	2.29	4.95	4.54	49.12	6.75	1.78	0.64
W2	28.23	3.02	4.00	4.87	52.43	5.55	1.34	0.56
WB1	26.68	1.67	1.52	1.75	62.03	4.47	1.52	0.36
WB2	26.14	1.08	1.81	2.33	63.15	3.98	1.17	0.34

## Data Availability

The original contributions presented in this study are included in the article. Further inquiries can be directed to the corresponding author.
